# Using model databases to determine dendritic distributions of *I*_h_ channels in oriens-lacunosum/moleculare hippocampal interneurons

**DOI:** 10.1186/1471-2202-13-S1-P41

**Published:** 2012-07-16

**Authors:** Vladislav Sekulic, Josh Lawrence, Frances K Skinner

**Affiliations:** 1 Department of Physiology, University of Toronto, Toronto, Ontario, Canada, M5S 1A8; 2 Toronto, Western Research Institute, University Health Network, Toronto, Ontario, Canada, M5T 2S8; 3 Department of Biomedical and Pharmaceutical Sciences, University of Montana, Missoula, Montana 59812, USA; 4 NIH COBRE Center for Structural and Functional Neuroscience, University of Montana, Missoula, Montana 59812, USA; 5 Departments of Medicine (Neurology), Physiology, and IBBME, University of Toronto, Toronto, Ontario, Canada

## 

The hippocampus is an important region of the brain that is critically involved in learning and memory, spatial navigation, and exploratory movements. Inhibitory interneurons are known to play a dominant role in the generation of population rhythms that are expressed during these behavioural states. One class of interneuron is the stratum oriens-lacunosum/moleculare (O-LM) cell that provides feedback inhibition and regulation of pyramidal cell activity. To better understand their particular contribution to hippocampal output, it is necessary to investigate their activities both intrinsically and in network contexts. To do this, the use of biologically grounded, multi-compartment, computational models is needed since they provide the ability to examine the simultaneous interaction of multiple conductances. However, due to the variability of experimental data, developing a database of models that collectively capture O-LM cell behaviour is required [1].

The goal of this research is to develop an O-LM model database to help determine balances of densities and distributions of conductances controlling O-LM cell output. Electrophysiological recordings of O-LM cells in mice have been obtained, and passive membrane properties for two morphological reconstructions of O-LM cells [2] have been fit using these datasets. Physiologically plausible ranges for the variation of conductance densities in the O-LM models have been specified, and simulations have been executed on the SciNet cluster to generate an O-LM model database. Analysis of the experimental datasets has been performed using the PANDORA software toolbox [3], the results of which have been applied to obtain a subset of appropriate O-LM models from the database.

In this work, our model database has been used to examine the distribution of hyperpolarization-activated cation currents (*I*_h_) in O-LM cell dendrites since this is currently unknown. *I*_h_ currents are known to have pacemaking roles, and it is important to determine whether *I*_h_ exists in dendrites as synaptic input onto O-LM cell dendrites would be duly influenced. A subset of the available models in the database has been compared and ranked to a subset of the electrophysiological data using PANDORA. The comparison is based on a quantitative distance metric between models and experimental traces using their extracted electrophysiological characteristics. To date, our comparisons and rankings indicate that models with *I*_h_ both in the soma and dendrite, rather than just in the soma, more closely conform to the experimental recordings. Our work therefore suggests that *I*_h_ could directly modulate incoming synaptic signals by its dendritic location, thus affecting the contribution of O-LM cells to hippocampal network rhythms.
